# Popular Lectures upon Medical Subjects

**Published:** 1873-06

**Authors:** 


### Editorial.

### Popular Lectures upon Medical Subjects.

We hereby acknowledge with many thanks invitation and cards of admission to Lectures which the following correspondence explains: If it is the beginning of an effort to educate the masses upon Medical Subjects, thus in some degree protecting from quackery and imposition, we think every well wisher of the race will take great interest in it. At present, even the better informed in general matters, are too profoundly ignorant of medical subjects to judge safely. It is not saying too much to affirm that in the United States with its popular system of education and general intelligence the people are too ignorant of Medicine and Hygiene to be safely intrusted with the care of themselves when suffering from disease. It is to be hoped that a better day is dawning for truth, and a worse one for falsehood and imposition.

——:o:——

NEW YORK, March 23d, 1873.

PROFESSOR R. OGDEN DOREMUS, M. D.

*Sir:*

Knowing that there is need of more accurate information, in the community, and in the country at large, on the subject of ANÆSTHESIA, we would respectfully request you to give us a Lecture, on the HISTORY AND PROPERTIES OF ANÆSTHETIC AGENTS, at such time and place as may suit your convenience.

We are your obedient servants,

WM. F. HAVEMEYER, F. H. HAMILTON, M. D., C. R. AGNEW, M. D.
J. MARION SIMS, M. D., FORDYCE BARKER, M. D., PETER COOPER, and a long list of well known citizens.

### PROFESSOR DOREMUS' REPLY.

"BELLEVUE HOSPITAL MEDICAL COLLEGE,"
*April* 21*st*, 1873.

HIS HONOR MAYOR HAVEMEYER AND OTHERS:

*Gentlemen:*

I feel highly honored with your polite request to lecture on the History and Properties of the Agents Employed to Produce Insensibility to Pain.

To accomplish your wishes more effectively I have ventured to solicit, and have obtained, the co-operation of Dr. J. MARION SIMS and Prof. FRANK H. HAMILTON, M. D., in presenting the *Historical* and *Surgical* department of this theme, limiting myself to the *Chemistry* of Anæsthetics.

At our invitation the Rev. HENRY WARD BEECHER has cheerfully consented to lend his valuable assistance on the occasion.

We have selected "Steinway Hall," on Wednesday Evening, May 21st, at 8 o'clock.

Trusting that the liberty I have taken may meet your approval,

I have the honor to remain,

Your obedient servant,

R. OGDEN DOREMUS.

---

### *ORDER OF EXERCISES.*

ORGAN VOLUNTARY.

HISTORY OF ANÆSTHESIA, . . . . . Dr. J. MARION SIMS.
CHEMISTRY OF ANÆSTHETICS, . Prof. R. OGDEN DOREMUS, M. D.
with Experiments.

*PRACTICAL APPLICATION OF ANÆSTHETICS IN SURGERY.*
Professor FRANK H. HAMILTON, M. D.

Address by Rev. HENRY WARD BEECHER.

*ORGANIST, Mr. CHARLES WALTER,*

Mayor HAVEMEYER will preside.

For this occasion Mr. WILLIAM STEINWAY has kindly tendered the use of STEINWAY HALL, Wednesday Evening, May 21st, 1873. at 8 o'clock.

